# Editorial: Harnessing the Participation of Dendritic Cells in Immunity and Tolerance

**DOI:** 10.3389/fimmu.2020.595841

**Published:** 2020-10-07

**Authors:** Silvia Beatriz Boscardin, Diana Dudziak, Christian Münz, Daniela Santoro Rosa

**Affiliations:** ^1^Department of Parasitology, Institute of Biomedical Sciences, University of São Paulo, São Paulo, Brazil; ^2^Laboratory of Dendritic Cell Biology, Department of Dermatology, Friedrich-Alexander University of Erlangen-Nürnberg (FAU), University Hospital Erlangen, Erlangen, Germany; ^3^Medical Immunology Campus Erlangen, Erlangen, Germany; ^4^Deutsches Zentrum Immuntherapie (DZI), Erlangen, Germany; ^5^Comprehensive Cancer Center Erlangen-European Metropolitan Area of Nuremberg (CCC ER-EMN), Erlangen, Germany; ^6^Viral Immunobiology, Institute of Experimental Immunology, University of Zürich, Zurich, Switzerland; ^7^Department of Microbiology, Immunology and Parasitology, Federal University of São Paulo, São Paulo, Brazil

**Keywords:** dendritic cell, DC subtypes, antigen presentation, dendritic cell vaccine therapy, dendritic cell activation

Dendritic cells (DCs) are cells of the innate immune system directly associated with the instruction and regulation of the adaptive immune response, thus bridging the innate and adaptive immune systems (1). DCs are the main antigen presenting cells (APCs), specialized in naive T cell priming into effector T cells. DCs recognize, internalize, process, and present antigens complexed to class I and II major histocompatibility complex molecules (MHCs), providing the three signals necessary for efficient T cell activation (2). Thus, DCs are essential for the induction of immune responses mediated by CD4^+^ and CD8^+^ T cells and, directly or indirectly, by B cells (3, 4).

However, DCs are not a homogeneous cell line, on the contrary, there are several subtypes that can be classified according to membrane markers and/or function. Basically, they can be classified into plasmacytoid and classical/conventional DCs (5, 6). Plasmacytoid DCs (pDCs) are specialized in the expression of type I interferons (IFN), responding quickly and efficiently to viral infections (7). Recent advances in the knowledge of their ontogeny, both in humans and in mice, are carefully reviewed by Musumeci et al., while Ali et al. review in detail the role of type I IFN production by these cells, but also by other cell types. Recent data indicate that several other cell types play a prominent role in the production of type I IFN, depending on the pathogen causing infection. Classical DCs (cDCs), on the other hand, are mainly associated with antigen presentation and T cell instruction. Most often, cDCs are subdivided into different subtypes based on the expression of various surface markers. However, a new classification according to their ontogeny has been proposed, as the functional identity of these cells is determined by their differentiation process (6, 8). A detailed review of the two main subtypes of cDCs (cDC1s and cDC2s), as well as their markers and location in different tissues, is presented by Backer et al. which, in addition, explore in great detail the origin, location, and role of splenic CD8α^+^Langerin^+^ cDC1s in the context of systemic infections and immunotherapy. Complementing the previous review, Prendergast et al. report that CD8α^+^Langerin^+^ cDC1s play an essential role in sepsis control (in a mouse model using BCG administered iv) as they activate CD8^+^ T lymphocytes and IL-12 production, which appear to be essential for the initial control of systemic bacterial infections. The role of cDC1s in anti-tumor immunity has not been fully clarified and Cancel et al. explore the latest literature on the subject providing evidence, mainly in animal models, that cDC1s have an important role in priming and/or sustaining the activation of both, T lymphocytes and NK cells. Human studies also point to a strong association between typical cDC1s signatures and a better prognosis for some types of tumors. The role of cDC1s in cancer, as well as the cross-talk between these cells and other DC subtypes and cell types, is discussed in detail by Noubade et al. who argue that understanding these interactions is important for the design of more effective DC-based cancer immunotherapies.

In this topic, the role of other cDC subtypes in different inflammatory, infectious diseases, and cancer was also explored. Tiburcio et al. analyze the interaction between DCs and *Leishmania* parasites, trying to elucidate the role of DCs in establishing the immune response in leishmaniasis and how this parasite subverts this response to establish itself. Furthermore, Nico et al. showed that a vaccine using *Leishmania (L.) donovani* antigens was able to prevent the dysfunctional migration of DCs seen in this animal model, and induce a protective response against this parasite. In the case of bacterial diseases, Richardson et al. demonstrated that dendritic cells derived from human monocytes (moDCs) are modulated by peptides derived from *Staphylococcus aureus* becoming more tolerogenic and consequently inducing regulatory T lymphocytes. The use of these peptides in vaccine strategies against autoimmune diseases appears very promising. Another molecule, derived from gram-negative bacteria, that has also been explored as an adjuvant in vaccination protocols, is flagellin. Flores-Langarica et al. demonstrate that lamina propria CD103^+^CD11b^+^ cDC2s respond to flagellin and are essential for the induction of T and B lymphocytes in the mucosa, especially inducing a Th2 type response. The effect of flagellin was further explored by Dos Reis et al. when moDCs from HIV-infected patients were treated with this compound. The treatment with flagellin suggested that this molecule was able to activate the inflammasome NAIP/NLRC4 and thereby promote the activation of moDCs derived from HIV^+^ patients. Still in the context of HIV infection, Schonfeld et al. found that the infection of moDCs with the sexually transmitted bacteria *Chlamydia trachomatis* followed by HIV infection was able to increase viral infection and to reduce the activation of cytotoxic T lymphocytes.

The role of different DC subtypes has been reviewed in the context of autoimmune diseases and pulmonary arterial hypertension (van Uden et al.). Although several DC subtypes were found to be involved, the exact functional role of each one, or of their interactions, in the development of these pathologies needs to be further defined. In fact, the crosstalk between different DC subtypes or with other cell types is extremely important, both in the steady state and in the context of inflammation. Grabowska et al. dedicate a detailed review on the interaction between CD169^+^ macrophages and DCs in the contexts of immunity and tolerance, presenting evidence that suggests that these cells greatly interact with each other, and that the cytokines produced by macrophages have an important impact on the activation of adaptive responses initiated by DCs against different pathogens. On the other hand, Wagner et al. evaluate the role of pDCs and 6-sulfo LacNAc-expressing monocytes (slanMo) in rectal cancer after neoadjuvant radiochemotherapy, and demonstrate that the benefits of this therapy in this particular cancer type may be related to increased infiltration of activated pDCs expressing IFNα and cytotoxic CD8^+^ T lymphocytes. Complementing these results, Ahmad et al. not only revise the role of slanMo in cancer, but also in other pathologies.

Most of the studies carried out to better characterize DCs and their functions and interactions have exploited either animal models or adult human samples. However, in recent years, different groups were dedicated to study the functional differences between the DC subtypes found in neonates and in adult individuals. Papaioannou et al. review such efforts and speculate on strategies that could be applied to promote neonatal immunity.

The capacity of DCs and other APCs in sensing the environment in which they are situated allows them to respond, or not, to different external stimuli. The ability of DCs and other APCs to respond to different sugars has been highlighted for the induction of immunity as well as tolerance. Lubbers et al. present a detailed review of how signaling through receptors that bind sialic acid is important in inducing immune tolerance, and how this knowledge could be utilized to develop strategies to treat autoimmune diseases or allergies. Another pathway capable of suppressing DC functions is that of adenosine (Ado), a metabolite of extracellular ATP (which acts as a danger signal in the context of inflammation). Silva-Vilches et al. present a careful review of how extracellular Ado suppresses the functions of DCs previously activated by the presence of extracellular ATP. Another important contribution was made by Han et al. who suggested that deficiency in Fas signaling in DCs increases allergy by inducing pulmonary inflammation mediated by the activation of a potent Th2 response. Other articles have explored not the signaling pathways, but rather the role of different transcription factors in DC activity. Sondergaard et al. and Tel-Karthaus et al. evaluate the role of DC-SCRIPT and Nur77 transcription factors, respectively, in human moDCs, demonstrating that both control the activation and presentation capacity of these cells, thereby modulating the activation of immune responses. The transcription factor Zeb1, on the other hand, seems to mediate the production of IL-6, IL-10, and IL-12 by cDC1, thus inducing a pro-inflammatory response (Smita et al.).

The success of DCs in antigen presentation and T cell priming can be partly explained by their remarkable ability to efficiently present internalized exogenous antigens in MHC I molecules. This phenomenon is known as cross-presentation. Through this process, DCs (especially the cDC1 subtype) are able to activate CD8^+^ T lymphocytes that play an important role during the course of the immune response, especially in viral infections and tumors. Three reviews discuss our current knowledge on the pathways that lead to efficient cross-presentation by DCs, from the acquisition of the exogenous antigen to its processing and peptide loading in the MHC I. Embgenbroich and Burgdorf discuss the two main cross-presentation pathways (the endosome-to-cytosol and the vacuolar pathways), while Gros and Amigorena emphasize what is currently known about the mechanisms for antigen export to the cytosol. Finally, Montealegre and van Endert debate in great detail the differences in the recycling pathways between DCs and non-antigen presenting cells. Despite some controversies in the field, these authors elegantly present an overview of the state-of-the-art of this very important phenomenon in DC biology.

Their incredible ability to modulate immune responses transforms DCs into ideal targets for manipulation. Once it was demonstrated that functional DCs could be obtained from peripheral blood monocytes (moDCs) (9), the next logical step was to produce these cells, load them with target antigens, induce maturation and administer them as vaccines. The first attempts to use autologous DCs loaded with antigens were made in patients with B-cell lymphomas more than two decades ago (10). Patente et al. review the clinical application of this technology, especially in cancer patients. In complement Huber et al., not only elaborate in detail the results obtained with the use of autologous moDCs in the treatment of cancer, but also present the potential use of the other subtypes of cDCs in this setting. On the other hand, da Silva et al. review the progress obtained with the use of moDCs for the treatment of HIV infection. Despite the various advances in the use of autologous moDCs as vaccines, the fact that these preparations are laborious, time-consuming and extremely expensive have led to research aimed at directing antigens directly to the different DC subtypes *in vivo*. Several strategies were then devised and promising results have been obtained in animal models. Kroczek et al. describe the use of the chemokine (C motif) ligand 1 (XCL1) to target the antigen to its receptor (XCR1) in cDC1s, while Koerner et al. review the use of poly (D, L-lactide-co-glycolide) microspheres (PLGA MS) in directing antigens to DCs. Antigen targeting using monoclonal antibodies (mAbs) against DC surface receptors fused to proteins of interest has also been studied as a promising way to deliver the antigen of interest to these cells. Apostolico et al. and Antonio-Herrera et al. explore the use of different adjuvants in combination with the administration of chimeric mAbs to improve the induced immune response. Differently, Zaneti et al. direct the antigen of interest through a DNA vaccine that encodes a single-chain Fv antibody designed to be secreted and direct the fused protein to cDC1s. In all cases, activation of the immune system was observed.

The use of animal models to study DCs ontogeny or the effect of ablation of a specific subtype on the development of the immune response can be very useful. In this context, Iwabuchi et al. created a model of humanized mice that allowed them to study the effect of fms-related tyrosine kinase 3 ligand (Flt3-L) and granulocyte-macrophage colony-stimulating factor (GM-CSF) cytokines on the development of human DC subtypes, Moreover, Mattiuz et al. were able to generate mouse strains capable of specifically depleting cDC1s. Further studies using these models will be important to determine cDC1 function in different settings.

In conclusion, this topic presents articles that contribute to a broader understanding not only of DC biology, but also of the processes involved in the many functions that these cells play during steady-state or infections/inflammation.

## Author Contributions

SB wrote the text. SB, DD, CM, and DR reviewed and approved the final version of this Editorial. All authors contributed to the article and approved the submitted version.

## Conflict of Interest

The authors declare that the research was conducted in the absence of any commercial or financial relationships that could be construed as a potential conflict of interest.
